# Investigating the impact of weak geomagnetic fluctuations on pigeon races

**DOI:** 10.1007/s00359-021-01534-x

**Published:** 2022-01-28

**Authors:** Petr Jandačka, Hynek Burda, Jiří Ščučka

**Affiliations:** 1 7775 Company, Evžena Rošického 1062/3, 721 00 Ostrava-Svinov, Czech Republic; 2 Department of Game Management and Wildlife Biology, Faculty of Forestry and Wood Sciences, Czech University of Life Sciences, Kamýcká 129, 165 21 Prague 6, Czech Republic; 3 Institute of Geonics of the Czech Academy of Sciences, Studentská 1768, 708 33 Ostrava, Czech Republic

**Keywords:** Magnetoreception, Pigeons, Magnetoreceptor, Geomagnetic fluctuations, Homing

## Abstract

The existence of avian magnetic orientation has been proved by many experimental studies, however, evidence for the use of magnetic cues by homing pigeons remains controversial. To investigate magnetic orientation by homing pigeons, we analyzed the results of pigeon races relative to weak fluctuations in the geomagnetic field, assuming that such disturbances could impact navigational efficiency if based on magnetoreception. Whereas most of the previous studies recorded and analyzed vanishing bearing of individually released pigeons, we evaluated relative duration of the homeward flight (homing speed, as a proxy of navigational efficiency) and its dependence on specific geomagnetic indices in racing pigeons released collectively. Our analysis of orientation efficiency of about 289 pigeon races over 15 years suggested slight negative correlations between geomagnetic fluctuations and homing time. Although the interpretation of this finding is manifold and not clear, it suggests that natural magnetic variations or disturbances can affect the homing orientation performance of pigeons. We suggest that studying pigeon races may have a heuristic potential and since these races are regularly and frequently organized in many countries all over the globe, examining homing performance relative to a suite of environmental variables may be useful for exploring hypotheses about pigeon navigation.

**Supplementary Information:**

The online version contains supplementary material available at 10.1007/s00359-021-01534-x.

## Introduction

Domestic pigeons (*Columba livia domestica*) are notable for their excellent long-distance homing abilities. They are opportunistic navigators able to use different types of sensory tools according to available cues (Walcott 1996; Wiltschko and Wiltschko 2017). Experiments with homing pigeons or migratory birds are typically based on analysis of initial orientation: vanishing bearings of released birds in the field, or displayed preferred escape directions in Emlen’s funnel in the laboratory (Emlen and Emlen 1966). Although the migratory birds choose the direction of their migration route at the given place and given time, pigeons typically vanish in the respective homeward direction. Many studies on homing in pigeons have presented evidence consistent with geomagnetic orientation and investigated the putative magnetoreceptor (Keeton 1971; Wiltschko and Wiltschko 2003, 2017; Wiltschko et al. 2007).

Our current knowledge of magnetoreception of birds is based mainly on behavioral experiments testing two main hypotheses, the magnetite hypothesis and the radical-pair hypothesis (reviewed in e.g. Winklhofer 2010; Wiltschko and Wiltschko 2019). The magnetite hypothesis assumes that magnetite crystals interact with the geomagnetic field while the magnetic force opens/closes mechanosensitive ion channels of neurons (Cadiou and Mc Naughton 2010). Alternatively, the magnetite structures could work as electromagnetic oscillators in the inner ears of birds (Jandacka et al. 2015). The magnetite structures were indeed observed in many organisms and some of them were designated as magnetoreceptors (Shaw et al. 2015). In the case of magnetite structures observed in the avian upper beak (Fleissner et al. 2003; Hanzlik et al. 2000) that had been proposed as magnetoreceptors, their magnetoreceptive role was subsequently questioned on histological grounds (Treiber et al. 2012, 2013). The proposed magnetoreceptive role of iron-rich organelles discovered in sensory cells in avian ears (Lauwers et al. 2013) was likewise questioned by the same research laboratory (Malkemper et al. 2019). The cryptochrome hypothesis assumes that short-wavelength light-sensitive cryptochrome molecules in the retina act as a magnetomodulator of light (Ritz et al. 2000). Recently, the existence of an electroreceptor instead of magnetoreceptor was hypothesised for pigeon magnetoreception on the base of electrophysiological measurements (Nimpf et al. 2019).

One of the critical arguments supporting the existence of magnetoreception is the influence of magnetic field disturbances on spatial orientation. Indeed, even small natural geomagnetic fluctuations (induced by deformation of the magnetosphere by the solar wind) were shown to affect orientation. These fluctuations range from zero to hundreds of nanotesla (on the order of 1% of the total magnetic field). Natural geomagnetic fluctuations affected homing in pigeons (Keeton et al. 1974; Kowalski et al. 1988) and this effect was masked by a magnet fixed on the back of pigeons (Larkin and Keeton 1976). More recently, Schiffner and Wiltschko (2011) have found that magnetic fluctuations disrupted magnetic orientation in pigeons (with the correlation between selected orientation parameters and values of geomagnetic fluctuations reaching coefficient values of almost − 1). Several articles reported the sensitivity of birds to magnetic variations in nanotesla range. An anthropogenic megahertz electromagnetic noise with a magnetic component in nanotesla range influenced the orientation of European robins in a laboratory (Engels et al. 2014) and artificial megahertz magnetic variations in nanotesla range influenced the orientation of free-living migratory robins in the field (Wiltschko et al. 2015; Winklhofer et al. 2013).

In the present analysis, we evaluate the influence of geomagnetic fluctuations on the orientation of pigeons. In contrast to the above cited studies on pigeon navigation which typically experimented with dozens individual pigeons homing over tens of kilometers, we analyzed pigeon races, where thousands of pigeons are released simultaneously and the birds are homing over hundreds of kilometers. Our approach is based on a logical axiom that geomagnetic disturbances should negatively affect magnetic orientation and efficiency of homing which is then reflected in the duration of the inbound (homeward) flight. This means that we have not examined starting orientation (vanishing bearings) and we have not followed the trajectory of birds but have taken relative duration (average homing speed) as a proxy of homing efficiency. We assume that different results in pigeon races are caused particularly by the length of the trajectory (which may be straight or more or less zigzag) rather than by individidual differences in actual flight speed.

Our approach is, however, not fully new. Pigeon races and homing speed have been analyzed with respect to navigation abilities and their dependence on meteorological and space weather variables (Dornfeldt 1996; Li et al. 2016; Walcott 1991). The results of those studies were not consistent. Whereas at some times and some places there was a relationship between the homing performance of pigeons and sun spots or natural variability in the Earth’s magnetic field, this relationship was not universal. Whether this is a consequence of the birds using multiple cues, or whether it simply is the result of chance sampling of the data, remained unclear. Some studies showed interference with space weather during overcast but not during sunny weather. Also, the influence of the race length and its direction may play a role (Dornfeldt 1996; Walcott 1991).

We have not included the meteorological variables into our analysis, as they change in time and course and also with the altitude of the home flight, whereas, the space weather is regionally (i.e. over the range of 500 km) the same and its time characteristics can be easily retrieved from open access archives of geomagnetic observatories. Our assumption is that the meteorological variables (sunny versus overcast, wind speed and direction) do not correlate with the space weather and their values were most likely randomly distributed over the races analyzed. Hence, their effect is not systematically adding and can be filtered out by statistics.

## Methods

### Pigeons

**Fig. 1:**
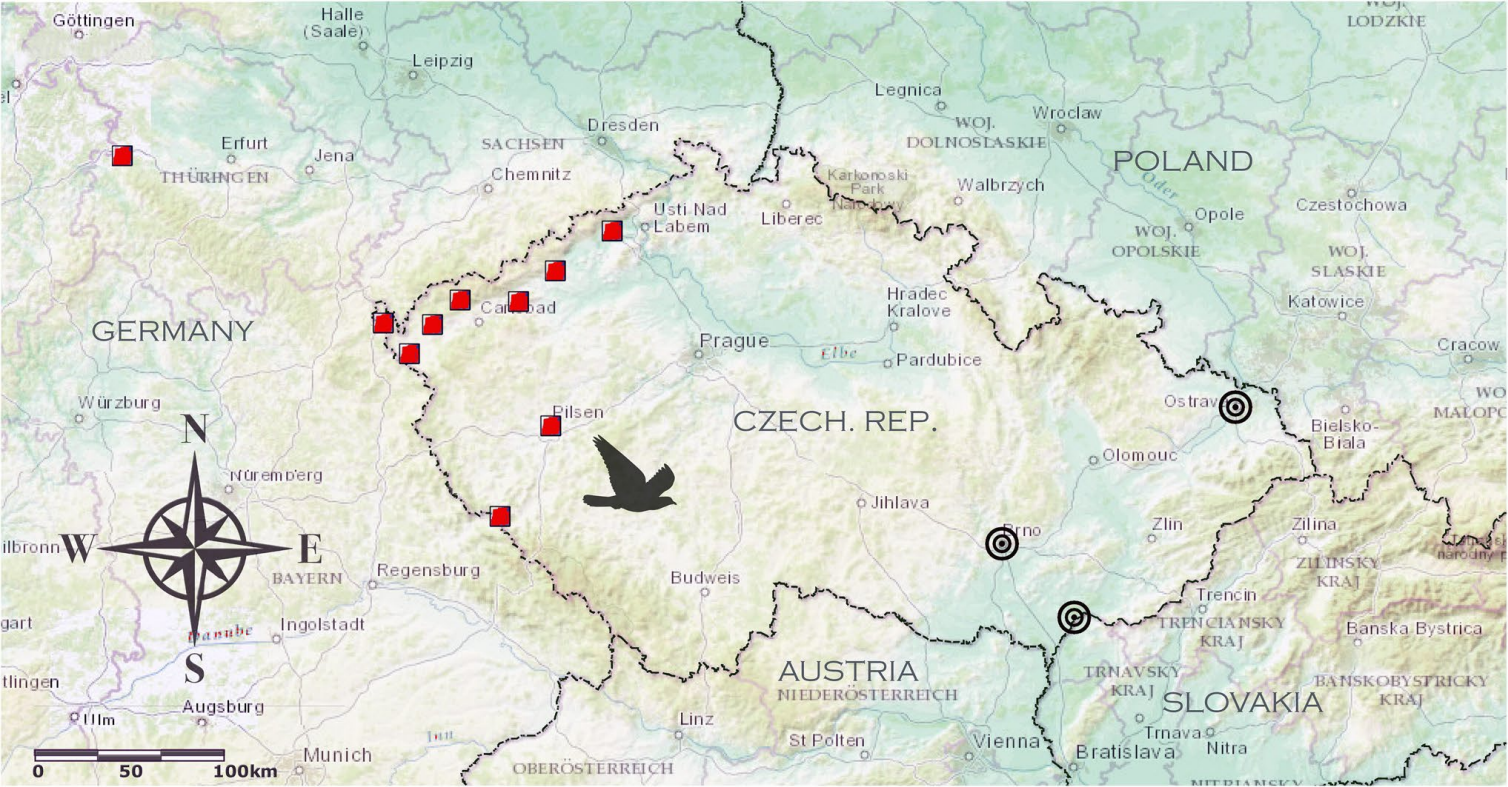
Map of the Czech Republic and Germany where the analyzed pigeon races took place. The start and finish points selected for this analysis are highlighted, with the red squares representing the start points and the rings representing the areas where the home lofts were situated. The distances varied between 330 and 550 km

For analysis, large datasets recorded during pigeon races were processed. The races were organized in the Czech Republic, Central Europe, with goals in the area of Ostrava, Brno and Hodonín, for 15 years (2000–2014). The release and start points are shown in Fig. 1—for details, see Supplemental information (SuI) file. The age of homing pigeons (*Columba livia*) was typically between 1 and 8 years and all pigeons were well-trained. The pigeon races are usually organized over distances from 200 to 1100 km. For our analysis, we picked the data from the middle-distance races, reaching from 330 to 550 km, because of the best data reliability. In total, we analyzed 289 pigeon races, with on average 2186 pigeons per race, from approximately 70 lofts, meaning that, typically, 20–80 pigeons originated from and flew to the same loft. Typical race procedure was as follows: pigeons bearing electronic chips or brass leg rings were registered electronically or manually 1 day before the race. After the registration process, the pigeons were loaded on a lorry and transported to the start point during the night. They rested at the start point for at least two hours before the start. Depending on the weather, the pigeons were released together (i.e. simultaneously) in the morning, usually between 5:30 and 9:00 a.m. of local time (i.e. 3:30–7:00 UTC). Typically after 5–20 min of orientation circling, they flew away. Pigeons were flying as singles or in pairs or groups and small flocks of different size. When they reached their home lofts, they were registered automatically in electronic clocks or manually using special mechanical clocks.

The resulting order of pigeon performances was determined based on their flight speed *v* = *s*/*t*, where *s* (m) is the direct distance determined from GPS positions of the start point and the home loft and *t* (s) is the flight time. The race finished when 20% of the fastest pigeons reached their lofts. The speed *v*_20_, which is the speed of the pigeon on the 20% position, is an important parameter for this article. For example, if 1000 pigeons participate in a race, the *v*_20_ parameter expresses the speed of the 200th pigeon.

### Geomagnetic indices

**Fig. 2:**
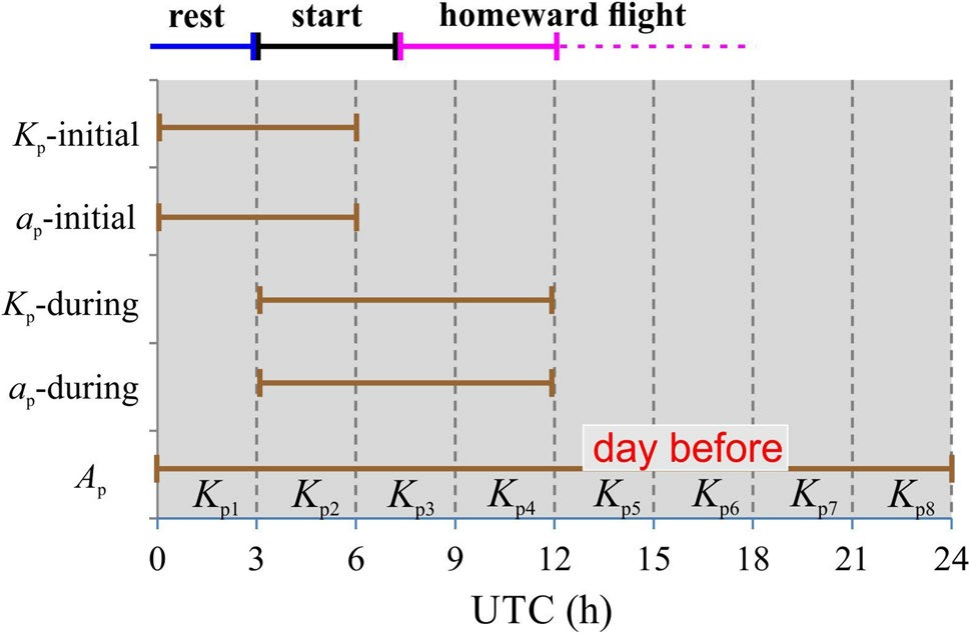
Geomagnetic indices and their time validity

The data of geomagnetic indices were taken from GeoForschungsZentrum, Postdam (see the SuI) and they are related to UTC (Coordinated Universal Time). We recorded the *K*_p_ index, which is a 3-hourly planetary geomagnetic index (*K*_p1_ 0–3 h, *K*_p2_ 3–6 h, etc., see Fig. 2) of activity, generated in Göttingen, Germany, based on *K* index from 12 or 13 stations distributed around the world (see the website: http://www.ngdc.noaa.gov/stp/GLOSSARY/). The *K*-index is a 3-hourly quasi-logarithmic local index of geomagnetic activity relative to an assumed quiet-day curve for the recording site, with the range from 0 to 9. The *K* index measures the deviation of the most distributed horizontal component of the geomagnetic vector. Typical 3-hourly fluctuation amplitudes reach tens of nanotesla. For example, in the case of *K* = 1, it is 5–10 nT, in the case K = 8 it is 330–500 nT. Since *K* index has no linear relation to real geomagnetic fluctuation, we transformed values of *K*_p_ index into *a*_p_ index using the function *a*_p_ = 0.0072·*K*_p_^5^ − 0.0173·*K*_p_^4^ − 0.0535·*K*_p_^3^ + 1.4851·*K*_p_^2^ + 0.9235·*K*_p_ + 0.1427. The *a*_p_ index is valid in the same day intervals as *K*_p_. Subsequently, *A*_p_ index was taken directly from Postdam website, which is a daily index calculated from *a*_p_ values by equation *A*_p_ = (*a*_p1_^2^ + *a*_p2_^2^ + *a*_p3_^2^ + *a*_p4_^2^ + *a*_p5_^2^ + *a*_p6_^2^ + a_p7_^2^ + *a*_p8_^2^)/8.

All *K*_p_, *a*_p_ and *A*_p_ indices express amplitudes of geomagnetic fluctuations. For this reason, we calculated own indices that are standard deviations (SD) of individual *a*_p_ values supposing that they could provide different information to the analysis. We selected some time intervals for *K*_p_, *a*_p_ and *A*_p_ indices concerning the schedule of races, see Fig. 2.

### Data processing

The correlation dependence between magnetic indices and the 1/*v*_20_ (min/m) parameter was computed using Matlab 7.0, Microsoft Excel and Statgraphics Centurion software. We used Pearson´s correlation coefficient *r*, which represents the degree of linear dependence between two sets of data and we subsequently determined two-tailed *P* value for determination of significance, where *P* ˂ 0.1 is valid for a 90% confidence level. Linear trends were calculated by the software from the least sum of squared deviations. Our criterion for a significant level of correlations was the boundary values ± 0.1. All correlation coefficients were computed from 289 points in graphs (289 races) and every single point in these graphs represents the movements of 2186 pigeons, which is the arithmetic mean per one race.

#### *Hypothesis*

The main hypothesis was that magnetic field fluctuations affect the magnetic orientation of pigeons, thus prolonging their homeward flight time, consistent with the previous reports (Kowalski et al. 1988; Schiffner and Wiltschko 2011). We do not examine here whether the putative effect of magnetic field fluctuation upon orientation is due to affecting the magnetoreception or due to unstable and thus unreliable magnetic cues. In the main orientation parameter *v*_20_ = *s*/*t*, the time has hyperbolic relation to the time *t*, which is inapplicable for linear correlation. The parameter *s* is constant since it is the direct distance between the start point and the home loft. Using transformation 1/*v*_20_ = *t*/*s*, a linear relationship between time and orientation parameter *v*_20_ can be obtained. Next, the parameter 1/*v*_20_ expresses a relative time. We expect a linear relationship between the geomagnetic indices and the 1/*v*_20_ parameter. We supposed that pigeons do not rely only on magnetic orientation ability, but they also use other navigation tools. Based on the earlier experimental papers on this topic where geomagnetic fluctuations prolonged vanishing intervals (Kowalski et al. 1988; Schiffner and Wiltschko 2011), we expected significant correlations for all tested indices vs relative homing time 1/*v*_20_, so we expected positive values of correlation coefficients. Therefore, the fluctuations of the geomagnetic field should disrupt the putative magnetoreceptor and worsen orientation and consequently also increase return time of birds. Duration of pigeon homeward flight is significantly influenced by weather (Dornfeldt 1996), which is the main source of strong variability in results, thus we expected a small but detectable effect of geomagnetic fluctuations given the relatively large sample size (*n* = 279 races).

## Results

**Fig. 3:**
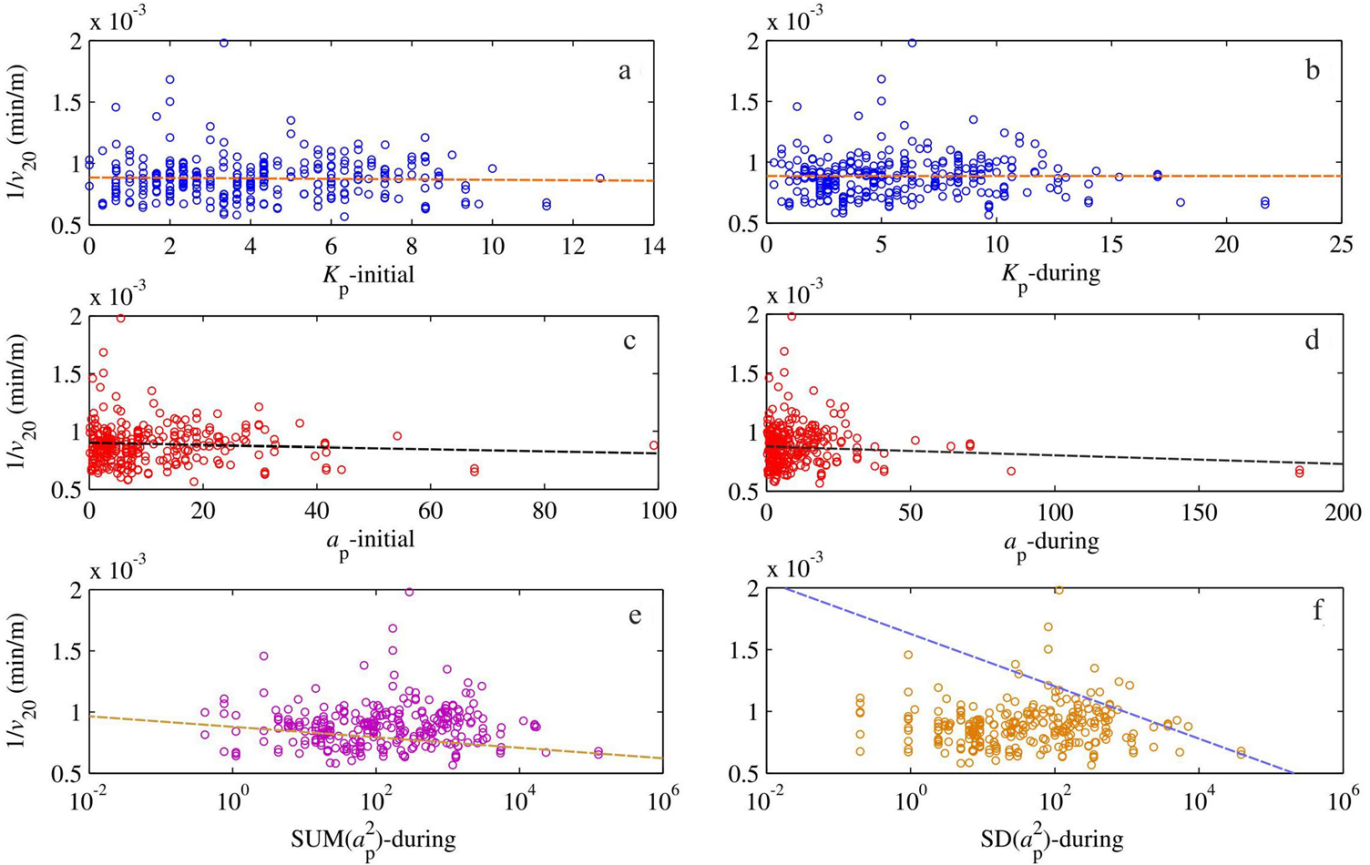
Examples of dependency between geomagnetic indices and 1/*v*_20_ parameter that represents the relative flight time (reciprocal speed) of pigeons, the performance of which equals the 20% position among all pigeons in the race. The dashed lines express the linear trend. The statistics were done taking into account the homing efficiency of 631,826 pigeons during 289 races, i.e. 289 points in graphs, where every single point represents mean homing efficiency of 2,186 pigeons. The individual figures represent dependencies of relative homing time 1/*v*_20_ on: **a** *K* index before and during start period, **b** *K* index during start and homing period, **c** *a*_p_ index before and during start period, **d** *a*_p_ index before and during start period, **e** sum of *a*_p_ indices during start and homing period and figure **f** represents the standard deviation of *a*_p_ indices during start and homing period. The figures **e** and **f** have logarithmic scales

All the selected indices were plotted with 1/*v*_20_ on graphs_._ Subsequently, Pearson´s correlation coefficient and two-tailed *P* value were determined. The results are presented in Table 1, while several graphical trends are shown in Fig. 3. Generally, the correlation coefficients are negative, but have values close to zero, most of them not exceeding the typical criterion boundary ± 0.1 for correlation. However, in the case of two indices, ∑(*a*_pi_^2^)-during and SD(*a*_pi_^2^)-during, there are stronger negative correlations. Their values are -0.114 and − 0.110, respectively. In statistics, where strong influences of random variables are present, those small correlations may express a real dependence and should not be overlooked. The strongest correlations were negative and were found out between 1/*v*_20_ and *a*_p_^2^, meaning that the stronger the fluctuations are, the shorter homing time was measured.

**Table 1:** Results of correlation between geomagnetic indices and 1/*v*_20_ parameter

*x*	*y*	*r* (Pearson´s)	Significance (*P* value)	Linear trend *y* = …
∑*K*_pi_-initial	1/*v*_20_	− 0.021	0.716	− 1.45·10^−6^·*x* + 8.89·10^−4^
∑*K*_pi_-during	1/*v*_20_	− 0.003	0.955	− 1.49·10^−7^·*x* + 8.84·10^−4^
*A*_p_-day before	1/*v*_20_	+ 0.066	0.260	+ 1.13·10^−6^·*x* + 8.72·10^−4^
mean(*a*_pi_)-initial	1/*v*_20_	− 0.046	0.432	− 6.38·10^−7^·*x* + 8.90·10^−4^
mean(*a*_pi_)-during	1/*v*_20_	− 0.085	0.150	− 7.65·10^−7^·*x* + 8.92·10^−4^
SD(*a*_pi_)-initial	1/*v*_20_	− 0.025	0.678	− 7.00·10^−7^·*x* + 8.86·10^−4^
SD(*a*_pi_)-during	1/*v*_20_	− 0.070	0.236	− 1.11·10^−6^·*x* + 8.89·10^−4^
∑*(a*_pi_^2^)-initial	1/*v*_20_	− 0.059	0.315	− 6.11·10^−9^·*x* + 8.87·10^−4^
∑(*a*_pi_^2^)-during	1/*v*_20_	− **0.114**	**0.054**	− 1.79·10^−9^·*x* + 8.86·10^−4^
SD(*a*_pi_^2^)-initial	1/*v*_20_	− 0.018	0.759	− 5.66·10^−9^·*x* + 8.84·10^−4^
SD(*a*_pi_^2^)-during	1/*v*_20_	− **0.110**	**0.062**	− 5.73·10^−9^·*x* + 8.86·10^−4^

∑ = sum, for example ∑*K*_pi_ − initial = *K*_p1_ + *K*_p2_; mean = arithmetic mean, for example mean(*a*_pi_)-during = (*a*_p2_ + *a*_p3_ + *a*_p4_)/3; SD = standard deviation, for example SD(*a*_pi_^2^)  − during = SD(*a*_p2_^2^, *a*_p3_^2^, *a*_p4_^2^), where SD = [∑(*a*_pi_–mean_*a*_p_)^2^/(*n*–1)]^0.5^. *P* ˂ 0.1 is valid for 90% confidence level

## Discussion

**Fig. 4:**
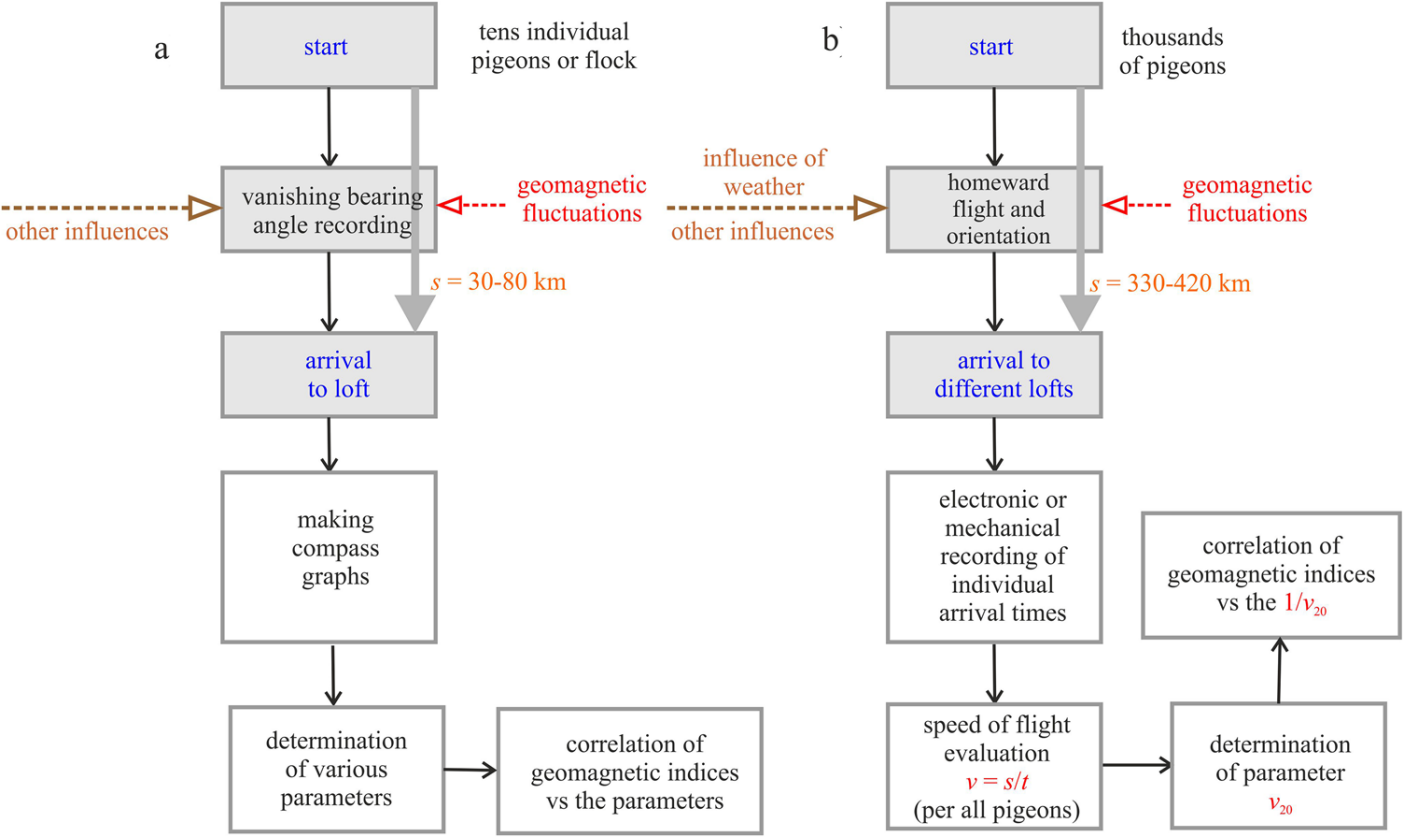
The difference in methodology between **a** typical previous analyses and **b** this analysis

Stronger geomagnetic fluctuations did not appear to decrease navigational efficiency in pigeon races, as measured by homing speed. This is in a seeming contrast with the previous conclusions demonstrating the disruptive effect of natural geomagnetic fluctuations upon the magnetic orientation of pigeons. The contradiction can be based on the different approaches and different methods of analyses, yet also on different interpretation of findings. These differences are summarized and illustrated in Fig. 4.

Apart from differences in the numbers of simultaneously released birds, the effect of which is not discussed here, the following relevant issues have to be considered. First, in most of the studies cited here (Keeton 1971; Keeton et al. 1974; Kowalski et al. 1988; Larkin and Keeton 1976), vanishing bearing (i.e. vector of initial orientation after release) was taken as a measure of magnetic orientation ability, while we have used relative duration of the entire homing flight as an evaluation criterion. However, there is no apparent reason to assume that the magnetic compass and magnetic map are switched on only during the initial phase of orientation. And indeed Schiffner and Wiltschko (2011) have argued that „*This continuing effect of magnetic fluctuations indicates that magnetic factors not only affect the beginning but remain an integral part of the pigeons’ navigational processes during the entire homing flight.*

Secondly, we used independent statistics based on homing parameters achieved in 289 trials by altogether about half a million pigeons that had flown several hundred kilometers toward home lofts with clearly no visual contact with their familiar home area. (Note that the visual horizon in the altitude of 100 m is 38 km.) The data have been collected by others for another reason than this analysis. In this respect the data collection was blind. Moreover, it can be assumed that the principal parameter 1/*v*_20_ is a valid criterion characterizing orientation efficiency (achievements) of pigeons and as such it has been also accepted by pigeon fanciers and pigeon races organizers. Interestingly, more of the correlation coefficients in our study are negative than we would expect by chance if there were no directional relationship between the magnetic indices and 1/v20.

Keeton et al. (1974) released pigeons under a sunny sky and tested correlations between *K* index and *mean vanishing bearing*, which was computed as vanishing bearing of a group of birds. The authors found that the fluctuations affected the initial orientation: the greater value of *K* index (i.e. higher magnetic disturbances) the higher the shift (deflection) of the mean vanishing bearing to the left (i.e. counterclockwise) from the expected homeward direction. The strongest correlation coefficient was − 0.561 (*N* = 16, *p* = 0.041). Note that high correlation coefficient (with higher *p* values) tends to be more likely with fewer data points. In additional experiments, the authors found that bar magnets (with masked the effect of geomagnetic fluctuations, while control groups of pigeons wearing brass bars instead of magnets were disrupted again (Larkin and Keeton 1976). Nevertheless, the results of the study as illustrated in the Figs. 1, 3 (Keeton et al. 1974) imply rather a different interpretation: namely, the higher the magnetic disturbances, the closer the mean vanishing bearing to the actual home dirrection—or, in another words,—the quieter the magnetic field, the larger the difference between the mean vanishing bearing and the actual home direction. Looking on the results of Keeton et al. (1974) from this point of view, they and our results are in agreement.

A similarly “positive” effect of a magnetic disturbance was observed in a more recent study by Mora and Walker (2012) who found that control pigeons vanished, on average, to the left of the home direction while a bar magnet attached at their head caused them to fly to the right of the control birds and thus actually corrected their homing heading. As a result, the pigeons carrying the magnet frequently vanished from the release site closer to the home direction than the control pigeons.

In another study, Kowalski et al. (1988) calculated correlations between *K*-indices and *mean vanishing bearing* (according to Keeton et al. 1974), *length of vanishing vector* (in circular statistics) and *duration of vanishing intervals*. Generally, they found that the geomagnetic fluctuations disrupted pigeons’ initial orientation. The fluctuations positively correlated with the duration of *vanishing intervals* and *mean vanishing bearings* (max. significant values were *r* =  + 0.684 with *n* = 12, *p* < 0.05 and *r* =  + 0.299 with *n* = 51, *p* < 0.05, respectively), while the parameter *length of vanishing vector* correlated negatively with the *K*-index (max. significant value was *r* = − 0.387).

More recently, Schiffner and Wiltschko (2011) analyzed GPS tracks of pigeons released north and south from their home lofts. The authors studied dependencies of specific orientation parameters during three homing phases (initial, departure and final) on *K*_p_ indices, *MV* indices (standard deviation of *a*_p_ indices) and *A*_p_ index. The specific orientation parameters were *median duration*, *angular deviation from mean*, *median steadiness* (based on the vector length) and *median flying speed*. The highest correlations were calculated for the *A*_p_ index versus *median duration* (*r* =  − 0.978), versus angular deviation from mean (*r* =  − 0.908) and *median steadiness* (*r* =  − 0.960), all of them for northward flight. The flying speed did not correlate. However, in our opinion, it could have been an effect of initial orientation delay combined with short distances, since during real pigeon races, the pigeons are significantly faster. Besides that it should be noted that the highest correlations were calculated only from 4 or 5 data points and relevance of the parameter *angular deviation from mean* is unclear since this parameter has no relation to homeward direction and might have been affected by inner mistakes. Its definition is *the deviation of the mean heading of the individual tracks recorded that day from the overall mean heading of all tracks recorded from the respective site*. Moreover, in the case of this parameter graphically plotted with *MV* index (see their Fig. 2 for departure phase), the angular deviation is high for zero values of *MV*, next the deviation is zero for middle values of *MV* and in the case of high values of *MV*, it increases again. Such a dependency seems to be contradictory.

Another interpretation is that larger distortions to the geomagnetic field tell pigeons to ignore magnetic map information and fly straighter or adopt other strategies that result in faster flight speeds. Perhaps magnetic map information does not help pigeons make faster progress toward their loft, but is used to ensure greater precision. That is, the map keeps pigeons from getting lost, but using it slows them down (N. Putman, personal comm.).

It is certain that the action force on magnetic elements (crystals, molecules, corpuscles) in the magnetoreceptors has linear relation with the geomagnetic index, whether one correlates the first (magnetic dipoles of single corpuscles) or the second power (two magnetized corpuscles) of the indices. Our finding that disturbances of the magnetic field actually improve homing achievement is thus paradoxical, yet it is consistent with the previous findings of effects of bar magnets attached to pigeons’ heads as described (but not interpreted in this way) by Keeton et al. (1974) and Mora and Walker (2012). This leads us to speculate that the magnetic sense may be based on electric, rather than on primarily magnetic, principle. Histological research should search also for tissue sensors reacting on electric voltage, not only for sensors based on the magnetic particles. In other words, a magnet statically attached to a pigeon does not affect a receptor system based on the electromagnetic induction, while magnetic field vibrations (caused by geomagnetic activity) do. As with other animals, the flight of a pigeon in a geomagnetic field itself causes electromagnetic induction in their bodies (as is commonly the case with machines on the ground), which can be detected by the mentioned hypothetic electric sensor. In addition, a vibrating geomagnetic field (activated by the sun activity) could just amplify this effect.

This hypothesis was recently advocated by the research team led by D. Keays (Nimpf et al. 2019). The authors proposed that movement of the head (head scanning) or even a small magnetic vector superimposed onto a larger fixed field would result in a changing magnetic stimulus and permit magnetoreception by electromagnetic induction. As the seat of this kind of magnetoreception the vestibular organ was proposed (Nimpf et al. 2019), consistently with the previous suggestions (Wu and Dickman 2011).

## Conclusions

Our analysis is consistent with the results of previous analyses showing that small natural magnetic variations or disturbances affect the homing performance of pigeons. However, and paradoxically, we consistently found negative correlations between two geomagnetic indices and relative homing time which means that the small geomagnetic fluctuations actually shorten the time of pigeon homeward flight, i.e. improve navigational efficiency. This finding is consistent with the results of previous experiments with bar magnets. We suggest that exploring environmental factors that correlate with timing in pigeon races could be informative of the sensory basis of navigation.

## Supplementary Information

Below is the link to the electronic supplementary material.

Supplementary file1 (XLS 335 KB)
